# Hypomagnesaemia As a Mortality Risk Factor in Protein-energy Malnutrition

**DOI:** 10.3329/jhpn.v29i2.7863

**Published:** 2011-04

**Authors:** Cahit Karakelleoglu, Zerrin Orbak, Candan Ferai Ozturk, Celalettin Kosan

**Affiliations:** ^1^Department of Pediatrics, Atatürk University Faculty of Medicine, Erzurum, Turkey; ^2^Department of Pediatric Endocrinology and Metabolism, Atatürk University Faculty of Medicine, Erzurum, Turkey

Sir,

Protein-energy malnutrition (PEM) is one of the leading causes of childhood morbidity and mortality in developing countries. The case-fatality rate for PEM was 28.4% despite hospitalization (1).

Magnesium (Mg) modulates vasomotor tone, blood pressure, and peripheral blood flow. Mg deficiency was shown to trigger vasoconstriction and enhance vascular endothelial injury (2). On the other hand, endothelin-1 (ET-1) is a potent vasoconstrictor peptide, produced by vascular endothelial cells (3). We investigated the effects of hypomagnesaemia on mortality and other mortality risk factors in patients with PEM.

The study included 25 children with PEM. Their mean age was 4.7+2.9 months. In the study groups, malnutrition was not related to malabsorption. Malnutrition was diagnosed using the Wellcome classification. All children had severe malnutrition without malabsorption (mean weight-for-height z-score was −3.1). Weights were measured using the same equipment by the same observer. Weights were measured to the nearest 10 g using a digital electronic instrument (Seca 727 Digital Baby Scale, serial interface RS 232, Seca Corporation, Vogel&Holke, Germany). Venous blood samples of the children were collected from the antecubital vein for serum Mg, and ET-1 measurements were obtained at admission. Blood samples were immediately centrifuged, separated, and stored at −80 °C before analysis. Serum magnesium levels of the 25 surviving and deceased patients were studied by spectrophotometrical xylidyl blue method (Olympus AU2700 autoanalyzer, commercially-available Boehringer Mannheim System kit). Reference range was accepted as 1.71-2.5 mg/dL. ET-1 levels were studied with ELISA method (BioTEK Power-WXS, commercially-available kit; Catalog No. 900-022).

Mann-Whitney U tests and odds ratio were used for the comparison of means of each group and for ratios of each group respectively.

Informed consent was obtained from parents. The Ethics Committee of the Faculty approved the study.

Serum Mg and ET-1 levels of the patients with PEM were prospectively evaluated. During the follow-up period, four (16%) patients died within four days after admission. Although serum Mg levels were significantly lower in the patients who died, serum ET-1 levels were slightly higher at admission (Table). Serum Mg levels were lower than normal value in three (75.0%) of the four deceased and in six (28.6%) of the 21 surviving patients with PEM. The odds ratio (odds ratio=(3/1)/(6/15)=7.5) for mortality was 7.5 times higher in the malnourished children with hypomagnesaemia (n=9) than in the malnourished children without hypomagnesaemia (n=16). When the mean weight-for-height measurements of the deceased and the surviving patients were evaluated, no significant difference (p>0.05) was observed between them.

**Table.** Mean±SD and significance values for serum Mg and ET-1 levels in the surviving and deceased patients with PEM

Parameter	Surviving patients (n=21)	Deceased patients (n=4)	p value
Mg (mg/dL)	2,06±0,33	1,55±0,43	<0.05
ET-1 (pg/mL)	42,23±14,81	45,17±13,95	>0.05

ET=Endothelin; Mg=Magnesium; PEM=Protein-energy malnutrition; SD=Standard deviation

We found significant differences in serum Mg levels but not in serum ET-1 levels between the surviving and the deceased groups of patients with PEM. Serum Mg levels were significantly lower in the paediatric patients (68.8%) in the intensive care unit compared to the control group (12.0%) (p<0.001) (4). Furthermore, Mg intake from drinking-water was suggested to exert a significant protective effect on the risk of cerebrovascular disease and death from stroke (5). Mg is one of the most abundant ions in human cells, and its serum concentration is remarkably constant in healthy subjects. Although the measurement of serum Mg does not always reflect the overall status of Mg metabolism, serum Mg correlates well with intracellular-free Mg, the physiological active form of the elements (6). However, even small alteration in the extracellular Mg concentration can influence human organism and metabolism.

When ET-1 levels were investigated as a mortality risk factor, Kunz *et al*. reported that plasma ET-1 levels were slightly higher but not significant in haemodialyzed patients with cardiovascular history compared to haemodialyzed patients without cardiovascular history (7). Adversly, plasma ET-1 concentrations are strongly related to outcome after acute myocardial infarction (p<0.0001) and provide prognostic information independent of clinical and biochemical variables. On the other hand, an experimental study showed that magnesium deficiency was associated with increased ET-1 levels and with significant proaggregatory and coagulation alterations (8). When Serebruany *et al*. investigated alterations in various haemostatic factors as risk factors for survival after acute myocardial infarction (9), no significant differences was found in the ET-1 plasma concentrations during occlusion between the surviving and the non-surviving groups in this study, although plasma baseline ET-1 levels was slightly higher but not significant in the deceased swine (9). This result was similar to ours. However, results of a previous study showed that the mean serum ET-1 levels in the group with low magnesium levels were significantly higher than that of the group with normal magnesium levels (p<0.05) in malnourished children (10). This discrepancy in our results may be related to the low number of the deceased patients with PEM. On the other hand, it has been shown a release of TNFα and IL-1 at approximately one week after the administration of a low Mg diet in mice (11). Results of another study showed that hypomagnesaemia could stimulate endothelial cells to produce and release ET-1 (12). So, the sampling time may be also important.

Since the number of cases chosen was small in our study, additional studies on pathophysiology and clinical importance of hypomagnesaemia are needed. The findings of the present study calls for giving attention to hypomagnesaemia as a mortality risk factor in PEM.
